# CIN III Diagnosed following Surgical Termination of Pregnancy

**DOI:** 10.1155/2014/389151

**Published:** 2014-05-19

**Authors:** Ciara Mackenzie, Abiodun Fakokunde, Abha Govind, Delaram Kermani

**Affiliations:** Department of Obstetrics & Gynaecology, North Middlesex University Hospital, Sterling Way, London N18 1QX, UK

## Abstract

We present a case of a 30-year-old mother of four who was incidentally diagnosed with cervical intraepithelial neoplasia (CIN) III following surgical termination of pregnancy. Five years previously a routine smear test had shown mild dyskaryosis but was never repeated. She was referred to colposcopy and, underwent loop excision of the transformation zone (LLETZ) and subsequently vaginal hysterectomy. Without this incidental finding she would have undoubtedly developed cervical cancer. We discuss the deficiencies in current cervical cancer prevention strategies and termination of pregnancy services. We emphasise the importance of ensuring that patients with dyskaryosis are not lost to follow-up and we consider whether there should be clearer guidance on the value of histological examination of products of conception following termination of pregnancy.

## 1. Introduction

In 2010, there were 2851 new cases of cervical cancer in the UK making it the 12th most common cancer among females [1]. Human papillomavirus (HPV) is the main risk factor for cervical cancer, particularly HPV types 16 and 18. Although most HPV infections do not progress to CIN, studies suggest that without persistent HPV infection cervical cancer will not develop and hence it is a “necessary cause” [2].

HPV vaccination was introduced in the UK in 2008 for girls aged 12 to 13. The quadrivalent vaccine currently in use provides up to 98% protection against CIN and cervical cancer caused by HPV types 16 and 18 [3]. It also protects against HPV types 6 and 11 which cause 90% of genital warts [4]. This public health programme aims to greatly reduce the number of newly acquired HPV infections; however, many women became sexually active before the immunisation programme was instigated and are therefore still at risk of HPV infection and its sequelae.

A national cervical screening programme was introduced in the UK in 1998 in order to try and prevent cervical cancer by detection and early treatment of cervical dyskaryosis, CIN, and HPV. It is estimated that cervical screening saves around 5000 lives each year in the UK and can prevent 45% of cervical cancer in women in their 30s [5]. Like any screening programme high uptake is essential to the success of the programme. In England in 2007-8 the coverage rate was 78.6% [5], the lowest rate since the call-recall programme began. Consequently, almost one quarter of eligible women are either not invited or do not attend cervical screening. Although emotional barriers such as fear and embarrassment are often cited as reasons for poor uptake, studies have shown that practical barriers such as “not getting round to it,” inconvenience of appointments, and trust in the test itself play equally important roles [6].

With regard to termination of pregnancy, almost 190,000 abortions were performed in England and Wales in 2011, of which 53% were surgical. Although 96% of abortions were funded by the NHS, 61% were performed in the independent sector under contract [7]. There is no consensus on the value of sending products of conception for histological examination following uterine evacuation. Independent providers must ensure fetal tissue is disposed of in a dignified and respectful manner but are not obliged to send tissue for histological examination. In our trust, tissue is sent following all evacuations of retained products of conception (ERPC) and on the rare occasions when terminations of pregnancy are performed within the gynaecology department. However, independent providers performing terminations of pregnancy at our hospital have no such obligation. The argument for performing histological examination is to confirm clinical findings and to make unsuspected and unusual diagnoses such as ectopic pregnancy, molar pregnancy, or incomplete/failed termination. Others, however, believe that routine histological examination does not reliably confirm or correct the preoperative diagnosis and that better training of clinicians in uterine evacuation and the preoperative recognition of rarer conditions are more important [8, 9].

## 2. Case Presentation

A 30-year-old mother with learning difficulties initially presented requesting sterilisation. She was para 4+2 having had four normal vaginal deliveries and two early miscarriages for which one was managed conservatively (1999) and the other surgically (2009). Her last smear test was in 2008 and had shown mild dyskaryosis. A search of her smear history showed she had not undergone a subsequent follow-up smear test as recommended.

She underwent hysteroscopic sterilisation but unfortunately this occurred in the luteal phase and she presented to the emergency department two weeks later with right-sided abdominal pain. Urinary pregnancy test was positive. She proceeded to urgent ultrasound scan to exclude ectopic pregnancy. Transvaginal ultrasound revealed a viable intrauterine pregnancy of six-week gestation. Rather than continuing with the unwanted pregnancy she opted for surgical termination of pregnancy (STOP) and laparoscopic sterilisation which was performed two days later.

**Figure 1:**
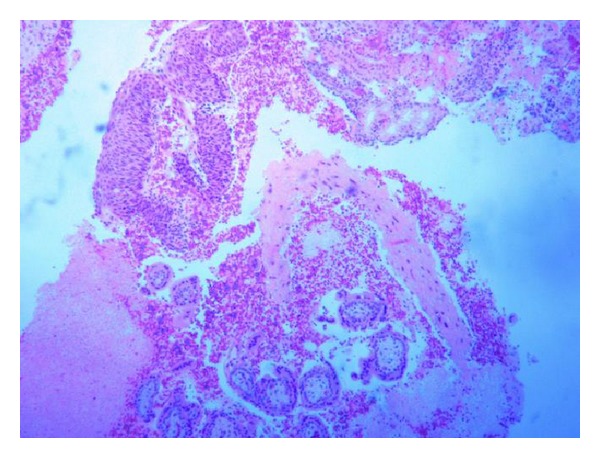
Products of conception with CIN II.

**Figure 2:**
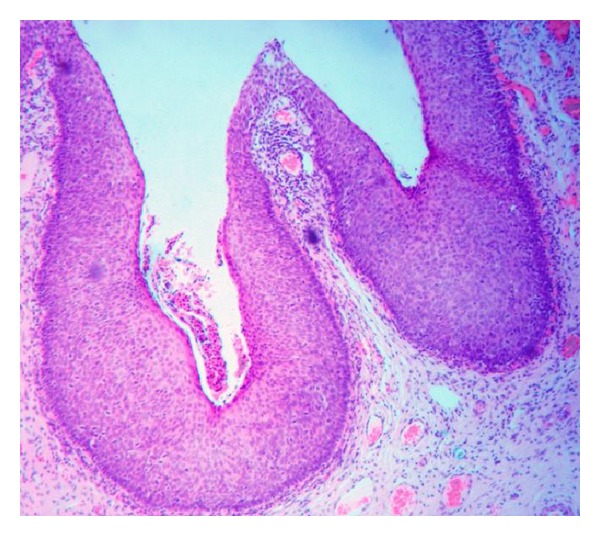
LLETZ with CIN III.

Routine histological examination following STOP revealed an incidental finding of moderate CIN II (Figure 1). She was therefore referred urgently to the colposcopy clinic and underwent colposcopy four weeks after the initial STOP. Biopsies taken at this initial appointment showed HPV and severe CIN III (Figure 2).

She was advised to have a LLETZ procedure and underwent this six weeks later. A large area of abnormality was visualised and removed piecemeal with ball diathermy to the edges. Histological examination showed CIN III and HPV. The pathologist was unable to comment on the completeness of the excision and therefore the slides were sent to our tertiary referral hospital to exclude microinvasion. This could not be excluded and after discussion at the multidisciplinary team (MDT) meeting the patient was recommended to proceed to vaginal hysterectomy.

She underwent uncomplicated vaginal hysterectomy five months after STOP and was discharged on day two postoperatively. Histological examination following vaginal hysterectomy showed CIN I focally extending to the resection margins. There was no evidence of high grade dysplasia or malignancy. She is not undergoing further treatment.

## 3. Discussion

We have identified two cases in the literature of CIN diagnosed at the time of uterine evacuation, one following ERPC for missed miscarriage [10] and one following termination of pregnancy at eight-week gestation [11]. This case adds to the evidence highlighting the importance of a routine histological examination following uterine evacuation be it following miscarriage or termination of pregnancy. Histological examination not only confirms the preoperative diagnosis, which for the majority of cases is normal products of conception, but also can provide new information such as evidence of molar pregnancy, incomplete evacuation, or cervical pathology. In this case the CIN was sufficiently advanced that without this incidental diagnosis, and especially given the patient's previous poor attendance for recall smear testing, she would have undoubtedly developed cervical cancer which would have been potentially devastating for her and her young family.

Of the 50,000 Essure (hysteroscopic sterilisation) procedures performed worldwide between 1997 and 2005, there were 64 unintended pregnancies reported to the manufacturer of which only eight cases were attributed to luteal phase pregnancy at the time of the procedure [12]. Our patient therefore only had a 1 : 6250 chance of undergoing hysteroscopic sterilisation whilst already pregnant. Having undergone sterilisation she represented to our hospital with pain and when found to have an intrauterine pregnancy was offered termination of pregnancy. This is a highly unusual sequence of events. Under normal circumstances if she had requested termination of pregnancy this would almost certainly have been performed by an independent provider without any histological examination performed.

Although we advocate histological examination following all uterine evacuations it is important to note that it is unclear what proportion of retained products contains cervical cells or what effect the suction aspiration has on the cervical epithelium architecture [10]. There is, of course, the potential that even with histological examination cervical abnormalities may not be detected. There is also the need to consider the cost-effectiveness of routine histological examination when the majority of cases will reveal normal products of conception.

Far more important in this case is that the patient did not have a repeat smear for five years following a finding of mild dyskaryosis. In order for cervical screening programmes to be effective not only must there be high population coverage but also abnormal findings must be acted on and recall smears repeated within an appropriate time frame. This case highlights the potentially devastating consequences of missed smear tests. Public health programmes such as HPV vaccination and cervical smear screening do not provide population coverage and it must be remembered that many women at risk of cervical cancer will not have accessed these programmes. Some young women may only access healthcare when they are pregnant or have a specific gynaecologic concern and in such circumstances opportunistic screening must be considered a necessity. Although generally cervical screening is not recommended in pregnancy due to inflammatory changes which make cervical cytology difficult to interpret, there are of course exceptions. In women who do not readily access healthcare, who have had a previous abnormal smear, or who are not up-to-date with their smears cervical screening must be offered regardless of pregnancy status. All clinicians, be they working in antenatal or gynaecology services within the primary, secondary, or independent sector, have a responsibility to check patients' smear histories and take opportunistic smears if and when necessary. The importance of this cannot be overemphasised.
